# Development and external-validation of a mechanistic dynamic nutrient-partitioning model for predicting growth and body composition in male guinea pigs (Cavia porcellus) of the Peru genotype

**DOI:** 10.14202/vetworld.2026.4085-4098

**Published:** 2026-09-18

**Authors:** Jaime Ricardo Rosero-Noguera, Juliana Carvajal, Sandra Lucía Posada-Ochoa

**Affiliations:** 1 GRICA Research Group, Faculty of Agrarian Sciences, University of Antioquia, AA 1226, Medellín, Colombia.

**Keywords:** body composition, dynamic modeling, growth prediction, guinea pig, mechanistic model, nutrient partitioning, Peru genotype, precision-feeding

## Abstract

**Background and Aim:**

Guinea pig production has considerable economic, nutritional, and cultural importance in Andean food systems. However, no mechanistic dynamic model is currently available to simultaneously predict growth and chemical body composition in this species. This study aimed to develop and externally validate a mechanistic nutrient-partitioning model for predicting live body weight (BW), empty BW, and chemical body composition in male guinea pigs (*Cavia porcellus*) of the Peru genotype.

**Materials and Methods:**

A mechanistic dynamic model integrating dry matter intake, maintenance requirements, protein and energy utilization, nutrient partitioning, and tissue deposition was developed and implemented in R. External-validation was performed using two independent datasets generated in Colombia. The first comprised longitudinal BW records from 184 male guinea pigs raised under commercial conditions, whereas the second included whole-body chemical composition measurements from 24 male guinea pigs. Predictive performance was assessed using Lin’s concordance correlation coefficient (CCC), coefficient of determination, root mean square error (RMSE), relative root mean square error, mean absolute error, and mean bias (MB).

**Results:**

The model predicted live BW with almost perfect agreement between observed and predicted values (mean CCC = 0.931; RMSE = 149.6 g; MB = −1.39 g). Final BW and empty BW were predicted with high accuracy (CCC = 0.99 and 0.98, respectively). Total body protein and ash showed almost perfect agreement (CCC = 0.91 for both), whereas body water showed substantial agreement (CCC = 0.70). In contrast, body fat was poorly predicted (CCC = 0.04; RMSE = 121.9 g) and was systematically underestimated. Prediction errors increased in animals weighing >1000 g, indicating reduced accuracy during the later growth phase.

**Conclusion:**

This is the first mechanistic dynamic nutrient-partitioning model developed specifically for guinea pigs. The model provides a biologically interpretable framework for simultaneously predicting growth and chemical body composition and may support nutritional assessment, precision-feeding, and decision-making in intensive production systems. However, parameters governing lipid accretion require further refinement before the model can be broadly applied across genotypes, sexes, and production environments.

## INTRODUCTION

The guinea pig (*Cavia porcellus*) was domesticated primarily as a food resource more than 4000 years ago in the Andean region, encompassing present-day Colombia, Peru, Ecuador, and Bolivia [1]. Today, guinea pig production plays an important economic, nutritional, and cultural role in these countries, particularly among rural and smallholder households, where it contributes to household income, local food systems, and the supply of high-quality protein in communities in which access to other livestock products is limited by economic or geographical constraints [2]. The increasing intensification of guinea pig production has consequently created a growing demand for feeding strategies that improve nutrient-use efficiency, growth performance, carcass composition, and overall production profitability.

Despite the increasing importance of guinea pig production, quantitative information regarding voluntary feed intake, nutrient utilization, maintenance and growth requirements, and the dynamics of chemical body composition remains limited [3]. Growth in guinea pigs has generally been described using empirical nonlinear equations that relate body weight (BW) to age [4]. Although these models effectively characterize phenotypic growth trajectories, they do not explicitly represent the underlying biological processes governing nutrient intake, maintenance requirements, nutrient partitioning, and tissue deposition [4–7]. Furthermore, empirical models generally predict BW without simultaneously estimating the accretion of individual chemical body components, including protein, fat, water, and ash.

To date, no mechanistic dynamic nutrient-partitioning model has been developed specifically for guinea pigs to simultaneously predict live BW, empty BW, and chemical body composition. Consequently, current modeling approaches provide limited insight into the biological mechanisms that regulate nutrient allocation among maintenance, protein deposition, lipid deposition, and overall tissue growth. This limitation restricts their application in evaluating nutritional strategies, optimizing nutrient utilization, and supporting precision-feeding programs under commercial production conditions. Therefore, developing a biologically based mechanistic model capable of integrating dry matter intake (DMI), nutrient requirements, protein and energy utilization, and body tissue accretion would represent a significant methodological advancement for growth prediction and nutritional management in guinea pigs [8–10].

We hypothesized that the growth and chemical body composition of male guinea pigs of the Peru genotype can be accurately predicted using a mechanistic dynamic framework that integrates DMI, maintenance requirements, protein utilization, energy utilization, and the differential accretion of body tissues. Therefore, this study aimed to develop and externally validate a dynamic nutrient-partitioning model to predict live BW, empty BW, and the accretion of protein, fat, water, and ash in male guinea pigs. Unlike conventional empirical growth models that predict BW alone, the proposed mechanistic framework reconstructs empty BW from its individual chemical components, thereby providing a biologically interpretable platform to support nutritional evaluation, growth simulation, and the future development of precision-feeding strategies in guinea pig production systems.

## MATERIALS AND METHODS

### Ethical approval

The animal procedures used to generate the chemical body composition dataset were reviewed and approved by the Institutional Animal Care and Use Committee of the University of Antioquia, Colombia (Approval Act No. 138, dated February 9, 2021). Animal management, euthanasia, sample collection, and whole-body chemical analyses were conducted in accordance with institutional and national guidelines governing the ethical use of animals in research.

The BW data used for growth-model validation were obtained retrospectively from routine production records collected at a commercial guinea pig production facility in 2018. These records were generated during standard farm management and did not involve additional animal handling, experimental treatment, or sample collection for the present study. Therefore, separate ethical approval was not required for the retrospective use of this dataset.

### Study period and location

The study used two independent datasets generated in Colombia. The growth-validation dataset comprised routine BW records collected in 2018 from a commercial guinea pig production facility. The body composition-validation dataset was obtained from an experiment conducted in 2021 at the University of Antioquia, Antioquia, Colombia. Model-development, simulation, statistical evaluation, and web application deployment were subsequently performed using the available experimental and production datasets.

### Study design

This study followed a mechanistic model-development and external-validation design. A dynamic nutrient-partitioning model was developed to predict live BW, empty BW (EBW), and the deposition of protein, fat, water, and ash in male guinea pigs. Model evaluation was performed using two independent datasets that were not used for model parameter estimation or calibration.

The first dataset consisted of repeated live BW measurements from 184 male guinea pigs raised under commercial production conditions. This dataset was used exclusively to evaluate the model’s ability to predict growth throughout the production period. The second dataset comprised 24 male guinea pigs of the Peru genotype subjected to whole-body chemical analysis. This dataset was used to evaluate predictions of EBW and the absolute amounts of body protein, fat, water, and ash. All simulations were conducted using the same equations and parameter values, without recalibration or dataset-specific adjustment, thereby ensuring an independent assessment of predictive performance.

### Model description

A mechanistic dynamic model was developed to predict growth and body composition in male guinea pigs based on nutrient intake and the efficiencies of protein and energy utilization. The model was based on nutrient-partitioning principles and simulated daily changes in EBW by tracking the differential deposition of protein, fat, water, and ash (Figure 1).

**Figure 1:**
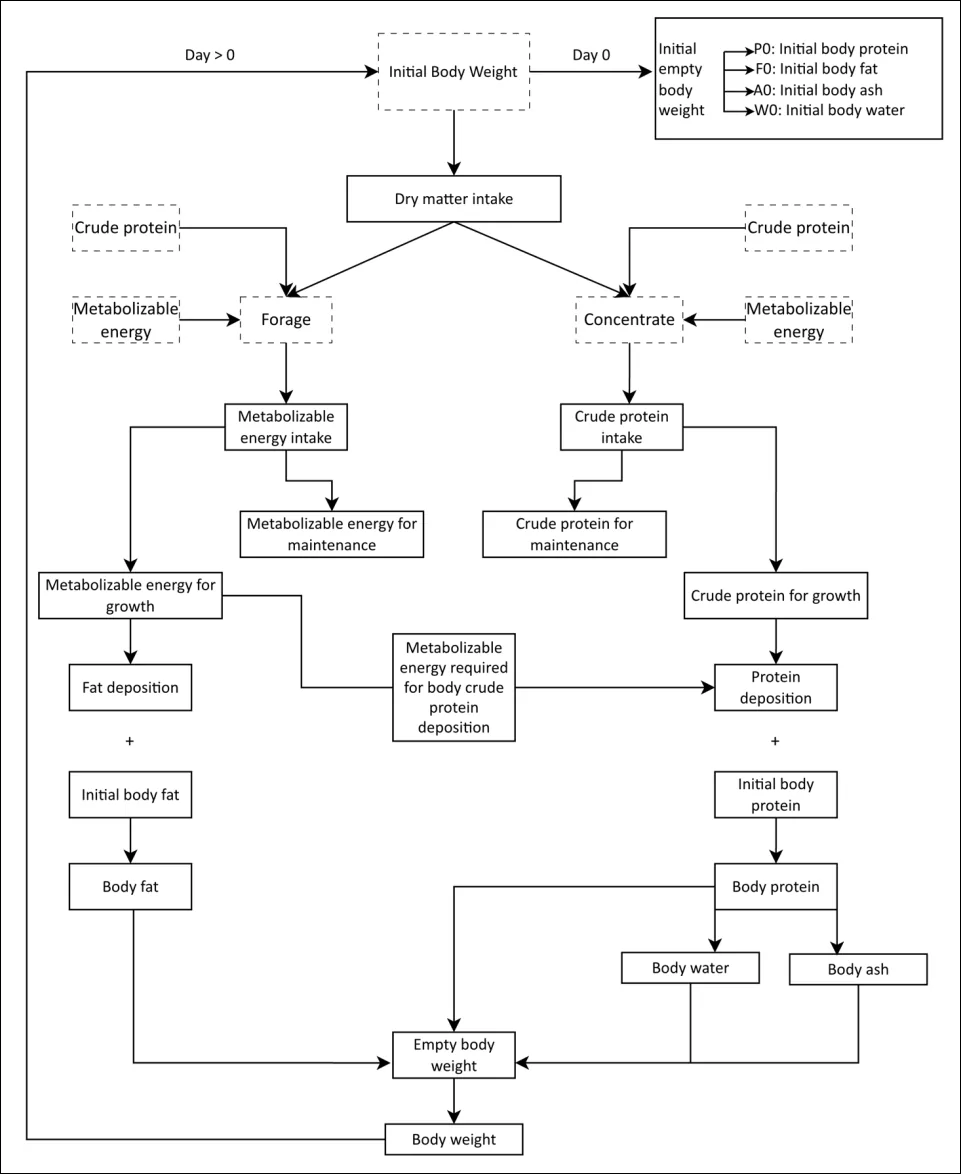
Schematic representation of protein and energy partitioning in guinea pigs for maintenance and growth. Dashed boxes indicate model inputs, whereas solid boxes represent state variables and model outputs. Arrows indicate the directional flow of variables within the model. EBW = Empty BW.

Initial live BW was used to estimate initial body composition through a series of empirical equations describing body protein, fat, water, and ash. Thereafter, BW and body composition were updated iteratively according to nutrient intake and the efficiencies of protein and energy utilization throughout the simulation period.

The model was implemented using R version 4.5.2 [11] and deployed as an interactive web application using Shiny version 1.12.1 [12]. The simulator is publicly available at https://rrnoguera.shinyapps.io/PrediCuy/ and can be used to simulate guinea pig growth under different dietary scenarios.

### Estimation of initial body composition

Initial live BW (BW₀, kg) was used to estimate EBW (kg) using the empirical relationship presented in the following equation [3]:

EBW = 1.0049 × BW₀ − 0.0675

Initial body protein (P₀), fat (F₀), water (W₀), and ash (A₀) were subsequently estimated using coefficients derived from previously reported experimental data [13]. These estimates defined the initial body composition at the beginning of the simulation (day 0):

P₀ = 0.2129 × EBW

F₀ = 0.00784 × EBW

W₀ = 0.7171 × EBW

A₀ = 0.0503 × EBW − 0.00008

### Nutrient intake

Daily DMI (kg/day) was estimated as a function of live BW (kg) using an allometric equation derived from previously published experimental data obtained from guinea pigs maintained under controlled feeding and growth conditions [3]:


\[
DMI=0.074\times {BW}^{0.6921}
\]

The model was designed to simulate forage-only, concentrate-only, or forage-concentrate diets. Forage DMI (DMI_f_) and concentrate DMI (DMI_c_) were estimated according to the dietary proportion of each feed component:


\[
{DMI}_{f}=DMI\times proportion of forage in the diet
\]

\[
{DMI}_{c}=DMI\times proportion of concentrate in the diet
\]

Daily crude protein intake (CPI, kg/day) and metabolizable energy intake (MEI, Mcal/day) were calculated from DMI and diet composition as follows:


\[
CPI=\left({DMI}_{f}\times {CP}_{f}\right)+\left({DMI}_{c}\times {CP}_{c}\right)
\]

\[
MEI=\left({DMI}_{f}\times {ME}_{f}\right)+\left({DMI}_{c}\times {ME}_{c}\right)
\]

where CP_f _and CP_c _represent the crude protein (CP) concentrations of forage and concentrate, respectively, whereas ME_f_ and ME_c_ represent their corresponding metabolizable energy concentrations.

### Nutrient requirements and partitioning

Maintenance requirements for CP (CP_mant_) and metabolizable energy (ME_mant_) were estimated as functions of EBW using the following equations [13,14]:


\[
{CP}_{mant}=6.32\times {EBW}^{0.75}
\]

\[
{ME}_{mant}=0.149\times {EBW}^{0.75}
\]

The amounts of CP and metabolizable energy available for growth were calculated as the difference between nutrient intake and maintenance requirements:


\[
{CP}_{growth} = (1000 \times CPI) - {CP}_{mant}
\]

\[
{ME}_{growth}=MEI-{ME}_{mant}
\]

Potential body protein deposition (PD_pot_) was estimated assuming a protein utilization efficiency of 62.9% [14]:


\[
{PD}_{pot}=0.629\times {CP}_{growth}
\]

The metabolizable energy required for protein deposition (ME_PD_) was estimated using the following equation [3]:


\[
{ME}_{PD}=0.0104204\times {PD}_{pot}+0.0042303
\]

The remaining metabolizable energy was assumed to be available for fat deposition (FD; ME_FD_):


\[
{ME}_{FD}={ME}_{growth}-{ME}_{PD}
\]

Body FD was calculated by dividing ME_FD_ by the energetic cost of lipid synthesis (0.01349 Mcal/g), which was derived from the energy content of 1 g of fat (0.00944 Mcal/g) and an assumed lipid deposition efficiency of 70% [15]:


\[
FD=\frac{{ME}_{FD}}{0.01349}
\]

### Updating body composition

After estimating daily protein and FD (PD_pot_ and FD, respectively), body composition was updated iteratively. Initial body protein, fat, water, and ash were defined at day 0 as P₀, F₀, W₀, and A₀, respectively. For subsequent iterations, the subscript *t* denotes the value of each variable on simulation day *t*.


\[
Pₜ₊₁ = Pₜ + ({PD}_{pot. t}/1000)
\]

*
Fₜ₊₁ = max[0, Fₜ + (FDₜ/1000)]
*

Body water and ash were subsequently estimated from body protein content using the following linear relationships:


\[
{W}_{t+1}=3.5694\times {P}_{t+1}+0.0014
\]

\[
{A}_{t+1}=0.2363\times {P}_{t+1}-0.00008
\]

EBW was calculated as the sum of all body components, whereas the final BW was estimated by applying a constant adjustment factor of 1.06 [3]:


\[
EB{W}_{t+1}={P}_{t+1}+{F}_{t+1}+{W}_{t+1}+{A}_{t+1}
\]

\[
B{W}_{t+1}=EB{W}_{t+1}\times 1.06
\]

The updated BW at the end of each simulation day was used as the input for the subsequent iteration. Model outputs included the temporal trajectories of BW, EBW, and the absolute masses of body protein, fat, water, and ash.

### Model assumptions

The proposed mechanistic model was developed to simulate growth and changes in chemical body composition in growing guinea pigs under controlled nutritional conditions. To maintain biological consistency while preserving model parsimony, several assumptions were adopted based on empirical evidence and established nutrient-partitioning principles.

The model assumes that growth results from the accretion of protein, fat, water, and ash. Accordingly, EBW is calculated as the sum of these components, and BW is derived from EBW using a constant conversion factor. Initial body composition is estimated from the animal’s initial BW, whereas body water and ash are predicted from total body protein using previously published equations.

DMI is assumed to depend primarily on BW, whereas the dietary concentrations of metabolizable energy and CP are considered constant throughout each simulation. Nutrient intake is first allocated to maintenance requirements, after which the remaining protein and energy are partitioned toward protein and fat accretion using constant utilization efficiencies.

The model does not explicitly account for the effects of genotype, environmental conditions, health status, or management practices on nutrient utilization. Therefore, its predictions should be interpreted within the biological and production conditions represented by the datasets used for model-development and external-validation. These assumptions were adopted to maintain model transparency while minimizing the number of empirical parameters required for simulation.

### Experimental datasets

Model evaluation was performed using two independent datasets generated under different production and experimental conditions in Colombia. The first dataset was used to evaluate the predictive performance of the model for BW during growth, whereas the second dataset was used to assess the accuracy of EBW and chemical body composition predictions. Neither dataset was used for parameter estimation or model calibration, thereby ensuring a completely independent external-validation process.

The first dataset comprised routine BW records collected from 184 male guinea pigs raised at a commercial production facility in 2018. The animals were weighed at birth and subsequently at 15, 28, 56, and 84 days of age. These data were used exclusively to evaluate the model’s predictive performance for BW throughout the growth period.

Under the routine management protocol, the animals were housed in group pens and fed a diet comprising fresh perennial ryegrass (*Lolium perenne*) and commercial concentrate at a forage-to-concentrate ratio of 70:30 on a dry matter basis. Diet composition remained constant throughout the production period and provided 16.8% CP and 2.62 Mcal of metabolizable energy/kg dry matter (Table 1).

The second dataset comprised 24 male guinea pigs of the Peru genotype from an independent experiment conducted at the University of Antioquia, Medellín, Colombia, in 2021. The animals were euthanized to determine EBW and the absolute amounts of protein, ether extract, water, and ash.

Chemical analyses were performed on the entire empty body after removal of the digestive contents to estimate chemical body composition. CP was determined using the Kjeldahl method (AOAC method 954.01), ether extract using Soxhlet extraction (AOAC method 920.39), dry matter by oven drying at 105°C (AOAC method 934.01), and ash by combustion in a muffle furnace at 550°C (AOAC method 942.05) [16].

**Table 1:** Descriptive statistics of observed BW in male guinea pigs at different ages under commercial production conditions (n = 184).

**Age**	**Mean (g)**	**Minimum (g)**	**Maximum (g)**	**SD (g)**
Birth	176.85	130	300	30.92
15 days	339.29	150	600	73.41
28 days	531.72	230	800	108.00
56 days	862.85	510	1300	139.79
84 days	1213.48	850	1800	122.55

BW = body weight; SD = standard deviation.

Data from this experiment had previously been used to estimate the net energy and net protein requirements of male guinea pigs, as reported by Tapie *et al.* [3, 14]. However, none of the animals in this dataset were used to estimate parameters or calibrate the equations incorporated into the proposed mechanistic model. Therefore, the dataset provided an independent framework for validating the model’s ability to predict EBW and chemical body composition (Table 2).

**Table 2:** Descriptive statistics of BW and chemical body composition, expressed as absolute and relative values, in male guinea pigs (n = 24).

**Variable**	**Mean**	**Minimum**	**Maximum**	**SD**
Initial BW (g)	403.75	323.00	514.00	55.12
Slaughter BW (g)	1069.46	713.00	1284.00	192.11
EBW (g)	995.50	621.00	1226.00	206.17
Empty body dry matter (g)	380.46	203.99	505.75	102.83
Protein (g)	208.35	126.82	276.65	46.19
Fat (g)	140.51	39.22	226.15	58.74
Water (g)	615.04	412.58	747.37	106.47
Ash (g)	49.23	29.97	65.37	10.92
Protein (% EBW)	20.91	18.44	23.34	1.24
Fat (% EBW)	13.46	5.80	18.60	3.76
Water (% EBW)	62.32	57.48	69.82	3.32
Ash (% EBW)	4.94	4.36	5.52	0.29

BW = body weight; EBW = empty BW; SD = standard deviation.

### Model evaluation

Model performance was assessed by comparing simulated outputs with observations from the two independent datasets. For each simulation, initial BW, dietary composition, and simulation duration were specified according to the corresponding experimental conditions. The model was then run at a daily time step to predict changes in BW, EBW, and the absolute amounts of protein, fat, water, and ash throughout the evaluation period.

Predicted BW values were compared with the observed measurements obtained from the commercial growth dataset, whereas predicted EBW and chemical body composition were compared with the corresponding measurements obtained through whole-body chemical analysis. Because neither validation dataset was used for model parameterization or calibration, predictive performance was evaluated exclusively through external validation. All simulations used the same equations and parameter values, without recalibration or dataset-specific adjustment.

### Statistical analysis

Model predictive performance was evaluated by comparing observed and simulated values for live BW, body protein (P), body fat (F), body water (W) and body ash (A). Following established approaches for evaluating deterministic models, model performance was assessed in terms of precision, agreement, prediction error and systematic bias.

The strength of the linear association between observed (* y *) and predicted (\(\widehat{y}\)) values was evaluated using the coefficient of determination (R^2^) obtained from simple linear regression. Overall agreement was assessed using Lin’s concordance correlation coefficient (CCC) [17], which combines precision, represented by the Pearson correlation coefficient (𝜌), with accuracy relative to the line of identity (\(y=\widehat{y}\)). The CCC was calculated as follows:


\[
CCC = \frac{2\rho {\sigma }_{y} {\sigma }_{ŷ}}{{\sigma }_{y}^{2} + {\sigma }_{ŷ}^{2} + {({\mu }_{y} - {\mu }_{ŷ})}^{2}}
\]

where 𝜌 is the Pearson correlation coefficient between observed and predicted values; σ_y_ and σ_ŷ_ are the standard deviations of observed and predicted values, respectively; and 𝜇_y_ and 𝜇_ŷ _are their respective means.

Based on previously proposed classification criteria [18, 19], CCC values between 0.81 and 1.00 were interpreted as indicating almost perfect prediction; values between 0.61 and 0.80 as substantial; values between 0.41 and 0.60 as moderate; and values between 0.21 and 0.40 as acceptable.

In addition, simple linear regression of predicted on observed values was used to estimate the intercept (\({\beta }_{0}\)) and slope (\({\beta }_{1}\)). Agreement with the line of identity was considered to improve as the intercept approached zero and the slope approached one. The coefficient of determination was used as an indicator of the proportion of variation in predicted values explained by observed values.

Overall prediction error was evaluated using the root mean square error (RMSE):


\[
RMSE = \sqrt{\frac{1}{n} \sum _{i=1}^{n}{{({y}_{i} -{ŷ}_{i} )}^{2}}}
\]

where \({y}_{i}\) and \({ŷ}_{i}\) denote the observed and predicted values for *i*th observation, respectively, and *n* is the number of paired observations.

Mean absolute error (MAE) and mean bias (MB) were also calculated as complementary indicators of residual magnitude and the systematic tendency to under- or overestimate observed values:


\[
MAE= \frac{1}{n} \sum _{i=1}^{n}{|{y}_{i} -} {ŷ}_{i}|
\]

\[
MB= \frac{1}{n} \sum _{i=1}^{n}{(}{ŷ}_{i }- {y}_{i} )
\]

Residuals were calculated as observed minus predicted values (\({y}_{i} -{ŷ}_{i}\)), whereas MB was calculated as predicted minus observed values (\({ŷ}_{i }- {y}_{i}\)). Therefore, positive MB values indicate model overestimation, whereas negative MB values indicate model underestimation. For ease of interpretation, RMSE was also expressed as a percentage of the observed mean (RRMSE).

All statistical analyses were performed in R version 4.5.2 [11] using the DescTools [20] and Metrics [21] packages.

## RESULTS

### Model performance for predicting growth

The predictive performance of the model for BW was evaluated by comparing observed and predicted values throughout the growth period. The validation metrics indicated strong agreement between the observed and predicted growth trajectories.

The mean CCC was 0.931, with individual values ranging from 0.789 to 0.998, indicating substantial to almost perfect agreement according to the established classification criteria [19]. The mean R² was 0.940 and ranged from 0.810 to 0.999.

The mean RMSE was 149.6 g, ranging from 28.6 to 280.9 g, whereas the mean RRMSE was 24.2%. The mean MAE was 111.5 g. The mean MB was close to 0 (−1.39 g), indicating no overall tendency of the model to underestimate or overestimate BW across the growth period (Table 3).

**Table 3:** Distribution of individual model performance metrics for predicting BW in male guinea pigs (n = 184).

**Metric**	**Mean**	**Minimum**	**Maximum**
CCC	0.931	0.789	0.998
R²	0.940	0.810	0.999
RMSE (g)	149.600	28.581	280.864
RRMSE (%)	24.200	4.600	56.040
MAE (g)	111.497	22.345	228.135
MB (g)	−1.390	−166.010	228.135

CCC = concordance correlation coefficient; R² = coefficient of determination; RMSE = root mean square error; RRMSE = relative root mean square error; MAE = mean absolute error; MB = mean bias.

Figure 2 shows the distributions of the model performance metrics calculated separately for each animal. Most CCC values were concentrated above 0.85, indicating consistently strong agreement between observed and predicted BW. The distributions of RMSE, RRMSE, MAE, and MB demonstrated variation in predictive performance among individual animals, although the overall MB distribution was centered near 0.

**Figure 2:**
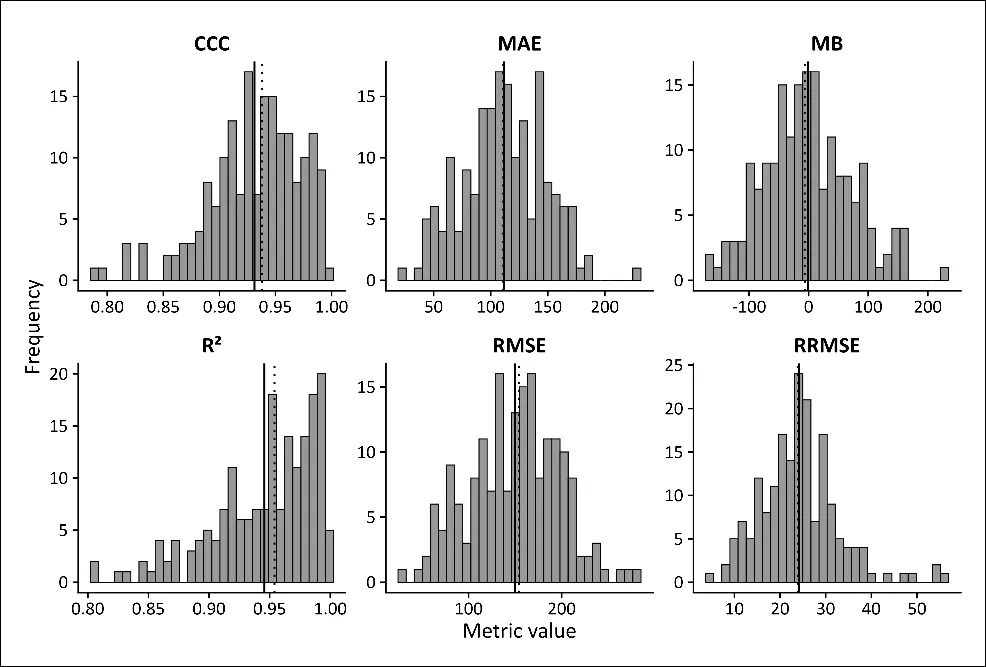
Distribution of individual model performance metrics for predicting BW in male guinea pigs. Solid and dashed vertical lines represent the mean and median, respectively. Each panel uses an independent scale to show the variability and distribution of predictive performance among animals. CCC = concordance correlation coefficient; R² = coefficient of determination; RMSE = root mean square error; RRMSE = relative root mean square error; MAE = mean absolute error; MB = mean bias.

Figure 3 shows the relationship between observed and predicted BW and the distribution of residuals relative to predicted BW. Most observations were located close to the line of identity, indicating strong agreement between observed and predicted values. However, the model tended to overestimate BW in animals weighing more than approximately 1000 g. Residual dispersion also increased at higher predicted BW values, indicating mild heteroscedasticity.

**Figure 3:**
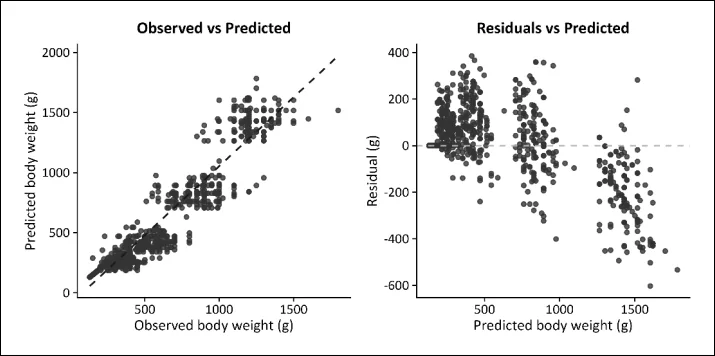
Relationship between observed and predicted BW (left) and residuals relative to predicted BW (right) in male guinea pigs. The diagonal line represents the line of identity (y = ŷ). Residuals were calculated as observed minus predicted values.

### Model performance for predicting body composition

The predictive performance of the model for chemical body composition was evaluated using the independent dataset of 24 male guinea pigs subjected to whole-body chemical analysis. Predictive performance differed among the evaluated body components (Table 4).

Final BW and EBW showed high coefficients of determination (R² = 0.99 and 0.98, respectively) and CCC values of 0.99 and 0.98, respectively, indicating almost perfect agreement between observed and predicted values. In contrast, final empty body dry matter showed moderate agreement, with a CCC of 0.45, despite its high R² of 0.94.

**Table 4:** External-validation metrics for predicting BW, EBW, empty body dry matter, and chemical body components in male guinea pigs (n = 24).

**Variable**	**Observed ** **mean**	**Observed ** **SD**	**Predicted ** **mean**	**Predicted SD**	**R²**	**CCC**	**RMSE ** **(g)**	**MAE ** **(g)**	**MB ** **(g)**
Growth variables									
Final BW (g)	1069.46	192.11	1071.65	191.65	0.99	0.99	6.22	4.92	2.19
Final EBW (g)	995.50	206.17	1010.95	181.11	0.98	0.98	38.24	29.17	15.45
Final empty body dry matter (g)	380.46	102.83	285.10	50.68	0.94	0.45	109.61	95.36	−95.36
Chemical body components									
Total body protein (g)	208.35	46.19	203.35	37.02	0.89	0.91	17.11	13.53	−5.00
Total body fat (g)	140.51	58.74	30.67	6.00	0.68	0.04	121.85	109.84	−109.84
Total body water (g)	615.04	106.47	725.85	132.14	0.96	0.70	116.23	110.81	110.81
Total body ash (g)	49.23	10.92	47.97	8.75	0.89	0.91	4.07	3.21	−1.26

BW = body weight; EBW = empty BW; SD = standard deviation; R² = coefficient of determination; CCC = concordance correlation coefficient; RMSE = root mean square error; MAE = mean absolute error; MB = mean bias.

Among the chemical body components, the model showed almost perfect agreement for total body protein and total body ash, both of which had CCC values of 0.91. The RMSE values were 17.11 g for total body protein and 4.07 g for total body ash. Total body water showed substantial agreement (CCC = 0.70), with an RMSE of 116.23 g and an MAE of 110.80 g. Total body fat showed the lowest agreement (CCC = 0.04), with an RMSE of 121.85 g and an MAE of 109.84 g.

Figure 4 shows the relationships between observed and predicted values and the corresponding residual distributions for the principal body variables. Observed and predicted values were closely aligned for BW and body protein. In contrast, body fat predictions were consistently lower than the observed values, as reflected by the displacement of the observations from the line of identity and the corresponding residual pattern.

## DISCUSSION

### Model performance for predicting growth

The present study demonstrates that a mechanistic nutrient-partitioning approach can accurately predict growth and most body composition components in growing guinea pigs under external-validation conditions.

**Figure 4:**
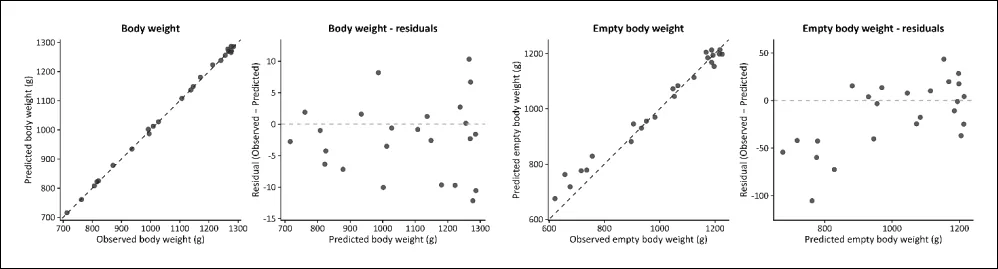
Relationships between observed and predicted values and the corresponding residual distributions for BW, EBW, body protein, and body fat in male guinea pigs. The diagonal dashed line represents the line of identity (y = ŷ). Residuals were calculated as observed minus predicted values.

Evaluation of BW dynamics demonstrated that the proposed mechanistic framework accurately reproduced the growth trajectory of guinea pigs maintained under controlled feeding and management conditions. The high concordance correlation coefficient (CCC = 0.931; Table 3) indicates strong agreement between observed and predicted values, confirming that the model adequately represents DMI and nutrient partitioning between maintenance and growth (Figure 3). From a biological perspective, the combination of high concordance and moderate prediction errors indicates that the model not only captured the overall growth trajectory but also maintained prediction errors within the range expected for experimentally validated mechanistic models [7, 22]. Furthermore, the negligible MB observed throughout the simulation period indicates no overall tendency to under- or overestimate BW, supporting the robustness of the model for simulating nutrient intake, maintenance requirements, and nutrient partitioning during growth [23, 24].

Although the overall MB was negligible, residual analysis revealed a systematic pattern associated with increasing BW. For animals weighing more than 1000 g, the model consistently overestimated BW, resulting in mild heteroscedasticity (Figure 3). This pattern reflects the compensation between slight underestimation at lower BW values and overestimation at higher BW values, producing an overall MB close to zero. Consequently, residual analysis provides additional insight into structural deviations that are not evident from global performance statistics alone [24, 25].

The discrepancy between observed and predicted values during the final stages of growth is likely associated with physiological changes as animals approach mature BW. As maturity is reached, an increasing proportion of nutrients is allocated to maintenance, thereby reducing the metabolizable energy available for tissue accretion [26, 27]. Simultaneously, muscle growth approaches its biological limit, reducing the efficiency with which nutrients are converted into BW gain [28]. Mechanistic models developed for monogastric species have demonstrated that the relationship between protein deposition and MEI above maintenance is curvilinear. Once the maximum capacity for protein accretion is reached, additional energy contributes predominantly to lipid deposition rather than further lean tissue growth, thereby altering nutrient partitioning and the growth response [29]. Therefore, the slight overestimation observed in heavier animals, together with the increased residual dispersion, suggests that although the model accurately describes the overall growth trajectory, it simplifies the physiological changes that occur during the late-growth phase.

### Model performance for predicting body composition

Validation using an independent dataset of 24 male guinea pigs confirmed that the proposed mechanistic model accurately predicted most body composition components during growth. The high coefficients of determination and concordance obtained for final BW (R² = 0.99; CCC = 0.99) and EBW (R² = 0.98; CCC = 0.98) indicate that the model provided accurate predictions of final BW and EBW while simultaneously representing the accretion of protein, fat, water, and ash (Table 4). Comparable predictive performance has been reported for mechanistic models developed for other livestock species [7, 23].

Total body protein was predicted with high accuracy, indicating that the mathematical representation of protein utilization efficiency adequately reflects the anabolic capacity of growing guinea pigs. This finding agrees with observations in other monogastric species, in which protein accretion generally exhibits lower biological variability than lipid accretion because it is regulated by more stringent homeostatic mechanisms [30, 31]. Likewise, total body ash was predicted with high concordance, supporting the proportional relationship assumed between body protein and skeletal mineralization. In growing monogastric animals, ash deposition is closely associated with skeletal development and therefore follows a pattern similar to lean tissue accretion [32].

Prediction of total body water showed substantial agreement, although the positive MB indicated systematic overestimation. This discrepancy likely reflects the model's assumption of a fixed protein-to-water ratio. Although body water is physiologically related to fat-free mass, the water-to-protein ratio changes during growth. In pigs, for example, this ratio gradually decreases as BW and body fat increase because of allometric changes in tissue composition and the progressive maturation of lean tissues [32]. Similar physiological changes may explain the systematic overestimation of body water observed in the present study.

Prediction of body fat represented the principal limitation of the current model. Lipid accretion is highly dynamic and responds to changes in energy availability, nutrient utilization efficiency, and physiological status. In mechanistic models for monogastric species, lipid deposition depends primarily on the metabolizable energy available above maintenance and on its interaction with the maximum capacity for protein accretion (Pdmax). Once protein deposition approaches its physiological limit, excess energy is redirected predominantly toward *de novo* lipogenesis, thereby increasing body fat without proportional increases in lean tissue [29]. The lower predictive performance observed for body fat suggests that the current model does not fully represent this hierarchy of nutrient partitioning in guinea pigs, possibly because the parameters governing energy allocation to lipid deposition have not yet been sufficiently characterized for this species.

The systematic underestimation of body fat suggests that the model allocates an insufficient proportion of available metabolizable energy to lipid deposition, particularly during the later stages of growth when protein accretion begins to plateau. Using constant values for the energetic cost of fat synthesis and the efficiency of lipid deposition, although common in mechanistic models, does not account for the biological variability associated with these processes. In pigs and other monogastric species, the efficiency of energy utilization for protein and lipid deposition varies according to nutrient source and nutritional status [29]. Therefore, the true efficiency of lipid deposition in guinea pigs, particularly in early-maturing genotypes, may differ from the fixed value assumed in the present model, thereby contributing to the observed discrepancy between predicted and observed body fat.

The simultaneous underestimation of body fat and overestimation of body water reflects the mass-balance constraint inherent to the model structure. Because predicted EBW is reconstructed within the model as the sum of protein, fat, water, and ash, underestimation of one compartment may be accompanied by overestimation of one or more of the remaining compartments, particularly body water. This behavior is consistent with findings from dynamic nutrient-partitioning models developed for other species, in which lipid is generally the most variable body component, whereas protein and ash exhibit substantially greater biological stability [33].

## CONCLUSION

The present study developed and externally validated a mechanistic nutrient-partitioning model capable of simulating growth and body composition dynamics in growing male guinea pigs under controlled nutritional conditions. External validation demonstrated that the model accurately predicted BW and EBW while providing high predictive accuracy for body protein and body ash. Prediction of body water showed substantial agreement, whereas body fat represented the principal source of prediction error. Overall, the model successfully reproduced the biological processes governing nutrient intake, maintenance requirements, and nutrient partitioning during growth.

The proposed model provides a biologically interpretable framework for predicting growth and body composition in guinea pigs using readily obtainable animal and dietary information. Because the model is based on physiological principles rather than empirical growth equations, it offers a valuable tool for evaluating feeding strategies, estimating nutrient requirements, and supporting nutritional decision-making in research and commercial guinea pig production systems. The framework also provides a foundation for future precision-feeding applications and simulation-based nutritional management.

A major strength of this study is the use of two completely independent datasets for external validation, ensuring that model performance was evaluated without parameter recalibration or dataset-specific adjustment. The mechanistic structure of the model enables direct biological interpretation of nutrient partitioning while maintaining relatively few empirical parameters, thereby enhancing model transparency and facilitating future refinement.

The model was developed and validated using male guinea pigs maintained under controlled nutritional and management conditions. Consequently, its applicability to females, different genotypes, alternative production systems, or varying environmental conditions requires further investigation. In addition, the reduced predictive accuracy for body fat indicates that the current representation of lipid accretion requires refinement, particularly during the later stages of growth when nutrient partitioning increasingly favors FD.

Future studies should incorporate genotype-specific information, environmental and management effects, and dynamic descriptions of lipid accretion to improve predictive performance under diverse production conditions. Additional validation using independent datasets representing different physiological stages, nutritional strategies, and production systems will further strengthen the applicability and robustness of the proposed modeling framework.

The proposed mechanistic nutrient-partitioning model represents a biologically consistent approach for simulating growth and body composition in growing male guinea pigs. The strong external-validation performance for BW, EBW, body protein, and body ash demonstrates its potential as a practical tool for nutritional research and production management. Further refinement of the lipid deposition component is expected to improve prediction of body fat and enhance the overall capability of the model for supporting precision nutrition and decision-making in guinea pig production.

## DATA AVAILABILITY

The data that support the findings of this study are available from the corresponding author upon reasonable request.

## GENERATIVE AI DECLARATION

The authors declare that generative artificial intelligence (AI) tools were used solely to improve language, grammar, and readability during manuscript preparation. All scientific content, data analysis, interpretation of results, and conclusions were developed and verified by the authors. The authors take full responsibility for the accuracy, integrity, and originality of the work presented, and no AI tool was listed as an author.

## AUTHORS’ CONTRIBUTIONS

JRRN and SLPO: Conceptualization, study design, and methodology. JRRN and SLPO: Software, validation, formal analysis, data curation, and visualization. JRRN and SLPO: Investigation and original manuscript preparation. JC: Data curation, verification of datasets, and interpretation of results. JRRN, JC, and SLPO: Manuscript revision and editing. All authors have read and approved the final version of the manuscript.
